# Aktueller Behandlungsstandard und Trends der Systemtherapie beim metastasierten hormonsensiblen Prostatakarzinom – Anwendung der Studiendaten in der Praxis

**DOI:** 10.1007/s00120-024-02410-7

**Published:** 2024-08-14

**Authors:** Mike Wenzel, Séverine Banek, Felix K. H. Chun, Philipp Mandel

**Affiliations:** Klinik für Urologie, Universitätsklinikum Frankfurt, Theodor-Stern-Kai 7, 60590 Frankfurt, Deutschland

**Keywords:** ARSI, Triplet-Therapie, Androgenrezeptorsignalweg, Chemotherapie, Prostatakrebs, ARSI, Triplet therapy, Androgen receptor signaling inhibitor, Chemotherapy, Prostatic neaplasms

## Abstract

**Hintergrund:**

Die Therapielandschaft des metastasierten hormonsensiblen Prostatakarzinoms (mHSPC) hat sich in den letzten Dekaden grundlegend von einer alleinigen Androgendeprivationstherapie (ADT) hin zu einer intensivierten Kombinationstherapien gewandelt.

**Fragestellung:**

Inwieweit haben die Daten der prospektiven Phase-III-Studien Einzug in den klinischen Alltag in der Behandlung des mHSPC innerhalb der letzten 5 bzw. 10 Jahre erhalten.

**Ergebnisse:**

Insgesamt konnten für die vorliegende Studie 1098 mHSPC-Patienten mit einem medianen Alter bei Metastasierung von 70 Jahre und einem medianen prostataspezifischen Antigen (PSA) von 43 ng/ml inkludiert werden. Signifikante Unterschiede zeigten sich bzgl. des PSA-Nadirs beim mHSPC nach Jahresstratifizierung. Ebenso zeigten sich signifikante Unterschiede bezüglich der eingesetzten Systemtherapien beim mHSPC und metastasierten kastrationsresistenten Prostatakarzinom (mCRPC; *p* < 0,001). Bezüglich der jährlichen Änderungsraten („estimated annual percentage changes“, EAPC) der letzten 10 Jahre zeigte sich ein signifikanter Abfall der ADT-Monotherapie von 85 % (2013) zu 29 % (2023, EAPC: −12 %, *p* < 0,001). Umgekehrter Weise zeigt sich ein signifikanter Anstieg von Substanzen zur Blockade des Androgenrezeptorsignalweges (ARSI) von 6 % in 2013 auf 55 % in 2023 (EAPC: +21,7 %, *p* < 0,001). Bezüglich der Docetaxel-Chemotherapie zeigt sich über die letzten 10 Jahre ein glockenhafter Verlauf von 8 % in 2013 zu 25 % in 2019 und einem Abfall auf 0 % in 2023. Der Anteil der Triplet-Therapien lag 2023 bei 16 %.

**Schlussfolgerung:**

Innerhalb der letzten 10 Jahre zeigt sich eine Adaptation der Phase-III-Studien zur intensivierten Kombinationstherapien beim mHSPC in der klinischen Realität mit dem häufigsten Einsatz von ARSI und Triplet-Therapie beim mHSPC.

## Abstract (en)

**Background:**

The treatment landscape of metastatic hormone-sensitive prostate cancer (mHSPC) has undergone fundamental changes in recent decades, moving away from the sole use of androgen deprivation therapy (ADT) and towards intensified combination therapies.

**Purpose:**

To what extent have the data from prospective phase III studies influenced clinical practice in the management of mHSPC over the past 5 or 10 years?

**Results:**

A total of 1098 mHSPC patients with a median age at metastasis of 70 years and a median prostate-specific antigen (PSA) level of 43 ng/ml were included in the present study. Significant differences were observed in terms of PSA nadirs in mHSPC after stratification by year of metastatic onset. Significant differences were also observed regarding systemic therapies applied in mHSPC and metastatic castration-resistant prostate cancer (mCRPC; *p* < 0.001). Regarding the annual estimated percentage change (EAPC) over the past 10 years, a significant decrease in ADT monotherapy from 85% (2013) to 29% (2023; EAPC: −12%, *p* < 0.001) was observed. Conversely, there was a significant increase in androgen receptor signaling inhibitor (ARSI) use from 6% in 2013 to 55% in 2023 (EAPC: +21.7%, *p* < 0.001). Regarding docetaxel chemotherapy, a bell-shaped pattern was apparent over the past 10 years, with rates increasing from 8% in 2013 to 25% in 2019 and decreasing to 0% in 2023. The proportion of triplet therapies was 16% in 2023.

**Conclusion:**

Over the past 10 years there has been an adaptation of intensified combination therapies for mHSPC in clinical reality, with the most frequent use of ARSI and triplet therapies.

## Einleitung

Über viele Dekaden hinweg war die vorherrschende Behandlungsmethode für Patienten mit metastasiertem hormonsensitivem (mHSPC) als auch metastasiertem kastrationsresistentem Prostatakarzinom (mCRPC) die alleinige Androgendeprivationstherapie (ADT; [1]). Nachdem erstmals im Jahr 2004 und folgend die ersten Kombinationstherapie aus ADT und Docetaxel sowie weitere Kombinationen für mCRPC-Patienten einen Überlebensvorteil zeigten, hat sich der Fokus der Forschung in den letzten 10 Jahren verstärkt auf das mHSPC verlagert [2, 3]. In dieser Zeit haben verschiedene Kombinationstherapien mit ADT plus Docetaxel-Chemotherapie oder Substanzen zur Blockade des Androgenrezeptorsignalweges (ARSI) wie Abirateron bei bestimmten Metastasierungsmuster wie „high volume“ nach CHAARTED oder „high risk“ nach LATITUDE einen signifikanten Überlebensvorteil gezeigt [4–6]. Zusätzlich konnten kürzlich für ARSI wie Apalutamid und Enzalutamid ein Überlebensvorteil unabhängig von der Metastasenlast und dem Zeitpunkt der Metastasierung (primäres vs. sekundäres mHSPC) nachgewiesen werden und somit stellen ARSI-Therapien den aktuellen Goldstandard in der mHSPC-Behandlung dar [7–9].

Die zuletzt präsentierten Daten zur sog. Triplet-Therapie aus ADT plus Docetaxel und Abirateron sowie ADT plus Docetaxel und Darolutamid zeigten zudem einen signifikanten Überlebensvorteil im Vergleich zur Standardbehandlung mit ADT und Docetaxel v. a. bei Patienten mit einer High-volume-Erkrankung [10–13]. Aktuell vergeht kaum ein internationaler Kongress, auf dem keine neue Daten zu Kombinationstherapien für das mHSPC und mCRPC vorgestellt werden. Offen bleibt, inwieweit die mHSPC-Kombinationstherapien der Phase-III-Studien auch wirklich Einzug in die klinische Praxis in der Behandlung des mHSPC gefunden haben. Entsprechend beschäftigt sich die vorliegende Arbeit mit der Frage nach den Trends der Anwendungen der Kombinationstherapien innerhalb der letzten 10 Jahre beim mHSPC aus Sicht der klinischen Behandlungsrealität.

## Material und Methoden

### Patientenkohorte

Für die vorliegende Studie wurden nach vorherigem Ethikvotum alle mHSPC-Patienten, welche mindestens einmal seit 2014 im Tumorboard des Universitären Centrums für Tumorerkrankungen Frankfurt (UCT) vorgestellt und diskutiert wurden, retrospektiv aus der metastasierten Prostatakarzinomdatenbank der Klinik für Urologie der Goethe-Universitätsklinikum Frankfurt, Deutschland, inkludiert. Alle Tumor- sowie Patientencharakteristika sowie onkologischen Verlaufsdaten wurden aus den vorliegenden elektronischen Patientenakten anonymisiert entnommen. Insgesamt qualifizierten sich somit 1098 mHSPC-Patienten für den Einschluss in die vorliegende Studie. Ein Großteil der Therapiesequenzen für Patienten im mHSPC und mCRPC wurde dabei durch die niedergelassenen Urologen und Urologinnen eingeleitet.

### Statistik und primäre Endpunkte

Die analytische Auswertung der Daten umfasste die Erfassung von Häufigkeitsverteilungen für kategorial gekodete Variablen sowie die Ermittlung von Medianen unter Einbeziehung der Interquartilsabstände (IQR) für kontinuierlich gekodete Variablen. Für die vorliegenden Tumor- und Patientencharakteristika wurden alle verfügbaren Daten innerhalb des Einschlusszeitraumes genutzt und eine Stratifizierung der Studienpopulation erfolgte entsprechend nach dem Jahr der Metastasierung (≤ 2006–2012 vs. 2013–2017 vs. 2018–2024).

Die primären Endpunkte der Studie waren die relativen Veränderungen des Anteils von De-novo- vs. sekundär metastasierten mHSPC-Patienten sowie die Therapieverteilungen im mHSPC über die letzten 5 bzw. 10 Jahre (seit 2013 bzw. seit 2018). Aufgrund des vornehmlichen Wandels der Systemtherapie im mHSPC innerhalb der letzten Dekade beschränkten sich die Trendanalysen lediglich auf die letzten 10 Jahre. Die Änderungen wurden statistisch mittels Log-linear-Regressionen erstellt, um die jährliche Änderungsrate („estimated annual percetange change“, EAPC) zu berechnen und graphisch darzustellen [14, 15]. Änderungen bei einem Signifikanzlevel von *p* < 0,05 wurden als statistisch signifikant gewertet. Sämtliche Analysen wurden mithilfe der Statistiksoftware R (Version 2023.12.1 +402) durchgeführt.

## Ergebnisse

Insgesamt konnten für die vorliegende Studie 1098 mHSPC-Patienten inkludiert werden (Tab. 1). Das mediane Alter bei der Metastasierung lag bei 70 (IQR: 64–76) Jahren mit einem medianen PSA von 43 (11–223) ng/ml. Der Anteil der Patienten mit Estern Cooperative Oncology Group (ECOG)-Status ≥ 2 lag an der Gesamtkohorte bei 6,5 %. Die Rate an De-novo-mHSPC-Patienten lag bei 60 % und eine primäre viszerale Metastasierung lag in 8,3 % der Fälle vor. Die Rate der Lokaltherapie bei Patienten mit Low-volume-de-novo-mHSPC stieg von 44 % (≤ 2006–2012) auf 66 % in 2018–2024 (*p* = 0,6) an.

**Tab. 1:** Vergleich von 1098 metastierten hormonsensitiven Prostatakarzinom (mHSPC)-Patienten, stratifiziert nach Jahr der Metastasierung des Prostatakarzinoms (PCa)

	*N*	Gesamt *N* = 1098^1^	Jahre ≤ 2006–2012, *N* = 165 (15 %)^1^	Jahre 2013–2017, *N* = 363 (33 %)^1^	Jahre 2018–2024, *N* = 570 (52 %)^1^	*p*-Wert^2^
Alter metastasiertes PCa	1029	70 (64, 76)	67 (61, 70)	70 (64, 75)	71 (64, 77)	< 0,001
PSA mHSPC	561	43 (11, 223)	15 (7, 150)	77 (17, 425)	40 (11, 175)	< 0,01
PSA-Nadir mHSPC	378	0,5 (0,1, 2,9)	2,0 (0,9, 4,5)	1,0 (0,2, 4,1)	0,3 (0,05, 2,0)	< 0,001
PSA-Abfall ≥ 99 %	316	153 (48 %)	4 (29 %)	27 (41 %)	122 (52 %)	0,095
PSA mCRPC	374	15 (4, 65)	30 (10, 64)	16 (4, 71)	13 (4, 64)	0,15
ECOG ≥ 2	796	52 (6,5 %)	4 (4,1 %)	31 (11 %)	17 (4,1 %)	< 0,001
Gleason Score	964	–				< 0,001
6–7	–	308 (32 %)	57 (45 %)	73 (23 %)	178 (34 %)	–
8–10		656 (68 %)	69 (55 %)	241 (77 %)	346 (66 %)	
Lokaltherapie De-novo-low-volume-mHSPC	160	94 (59 %)	4 (44 %)	22 (46 %)	68 (60 %)	0,6
Viszerale Metastasen mHSPC	902	75 (8,3 %)	8 (7,4 %)	21 (7,8 %)	46 (8,8 %)	0,8
High-volume-mHSPC	655	327 (50 %)	22 (54 %)	99 (58 %)	206 (46 %)	0,028
High-risk-mHSPC	669	364 (54 %)	25 (58 %)	110 (63 %)	229 (51 %)	0,022
De-novo-mHSPC	1076	645 (60 %)	73 (48 %)	225 (62 %)	347 (62 %)	< 0,01
Systemtherapie mHSPC	1064	–				< 0,001
Docetaxel	**–**	107 (10 %)	4 (2,5 %)	44 (13 %)	59 (11 %)	–
ADT mono/nmHSPC/nmCRPC		601 (56 %)	138 (86 %)	269 (77 %)	194 (35 %)	
ARSI		308 (29 %)	17 (11 %)	27 (7,7 %)	264 (48 %)	
Triplet		28 (2,6 %)	0 (0 %)	0 (0 %)	28 (5,1 %)	
Other		20 (1,9 %)	2 (1,2 %)	10 (2,9 %)	8 (1,4 %)	
Systemtherapie mCRPC	1098	–				< 0,001
ADT mono	**–**	71 (6,5 %)	30 (18 %)	36 (9,9 %)	5 (0,9 %)	–
Chemotherapie		132 (12 %)	35 (21 %)	44 (12 %)	53 (9,3 %)	
RLT		24 (2,2 %)	1 (0,6 %)	9 (2,5 %)	14 (2,5 %)	
ARSI		367 (33 %)	61 (37 %)	157 (43 %)	149 (26 %)	
Radium		32 (2,9 %)	8 (4,8 %)	23 (6,3 %)	1 (0,2 %)	
Keine/andere/NA		472 (43 %)	30 (18 %)	94 (26 %)	348 (61 %)	

*PCa *Prostatakarzinom,* PSA* prostataspezifisches Antigen, *mCRPC* metastasiertes kastrationsresistentes Prostatakarzinom, *ECOG* Eastern Cooperative Oncology Group, *ADT* Androgendeprivationstherapie, *nmHSPC/nmCRPC* nicht-metastasiertes HSPC/CRPC, *ARSI* Substanzen zur Blockade des Androgenrezeptorsignalweges, *RLT* Lutetium-Radioligandentherapie, *NA* unbekannt/Nichterreichen des mCRPC

^1^ Median (IQR), *n* (%)

^2^ Kruskal-Wallis rank sum test; Pearson’s χ^2^-Test; Fisher’s exact test

Nach Stratifizierung entsprechend der Diagnosejahre zeigt sich eine signifikante Zunahme im Alter der Patienten bei Metastasierung von 67 Jahren (≤ 2006–2012) zu 70 Jahren (2013–2017) und 71 Jahren (2018–2024, *p* < 0,001). Ebenso zeigte sich bei der Behandlung des mHSPC ein signifikant tieferer PSA-Nadir bei den beiden zuletzt behandelnden Kohorten (median 0,3 ng/ml für 2018–2024 und 1,0 ng/ml für 2013–2017) im Vergleich zu der eher historisch behandelten mHSPC-Kohorte (2,0 für ≤ 2006–2012, *p* < 0,001). Diese Unterschiede des absoluten PSA-Nadirs spiegelten sich auch im relativen PSA-Abfall ≥ 99 % wider, ohne ein Erreichen des Signifikanzniveaus: 52 % vs. 41 % vs. 29 % für die Jahre 2018–2024 vs. 2013–2017 vs. ≤ 2006–2012 (*p* = 0,095). Die Rate an High-volume- nach CHAARTED-Kriterien sowie High-risk-mHSPC-Patienten nach LATITUDE zeigte sich in den aktuell behandelnden Patienten am geringsten.

### Veränderungen im Metastasierungszeitpunktes

**Abb. 1:**
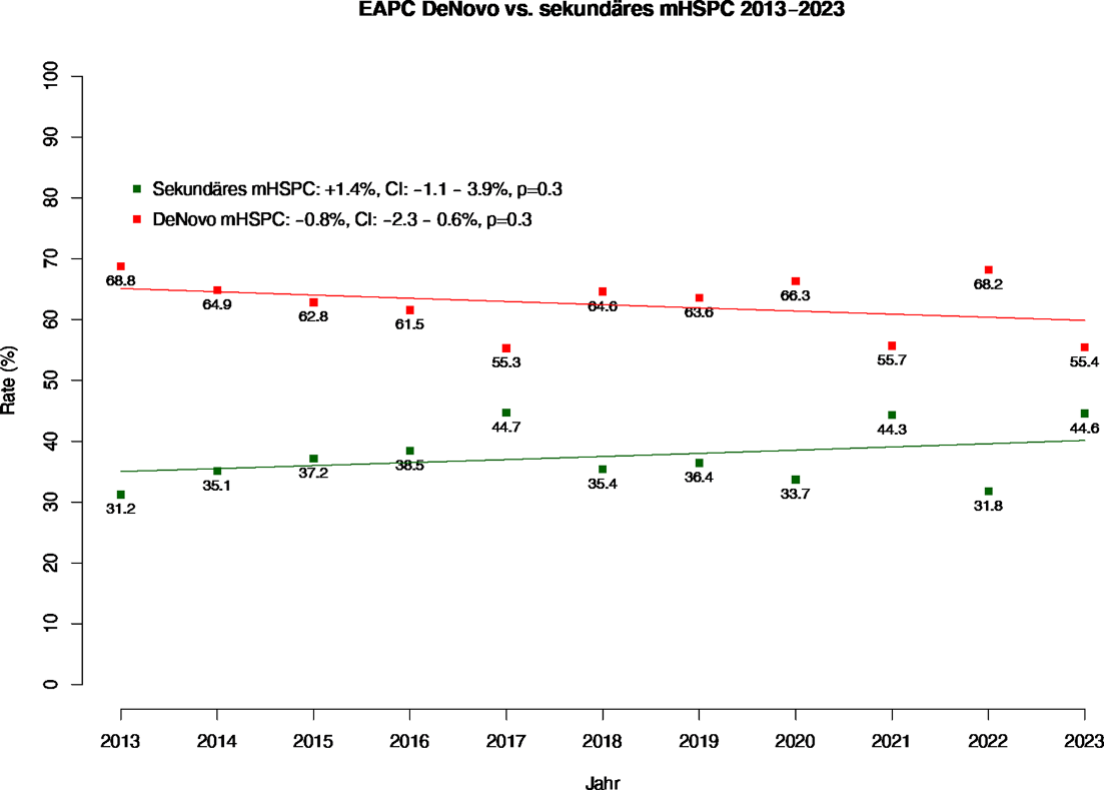
Trends von De-novo- vs. sekundär („estimated annual percentage change“, EAPC) metastasierte hormonsensitive Prostatakarzinome (mHSPC) im Zeitraum 2013–2023

Bei Rate der De-novo-metastasierten mHSPC-Patienten zeigt sich eine signifikante Zunahme von 48 % (Jahre ≤ 2006–2012) auf 62 und 62 % für die Jahresintervalle 2013–2017 sowie 2018–2024. In der Darstellung der EAPC-Trendanalysen (Abb. 1), zeigt sich über die letzte Dekade (2013–2023) keine statistisch signifikanten Veränderungen im Verhältnis von De-novo- vs. sekundär metastasierten mHSPC-Patienten.

### Verwendung und Trends der Systemtherapien

**Abb. 2:**
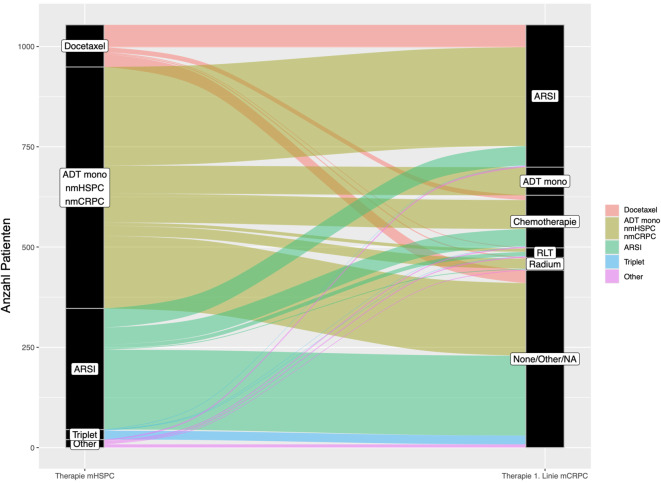
Sankey-plot-Darstellung der Therapielinien beim metastasierten hormonsensitiven Prostatakarzinom (mHSPC) und des metastasierten kastrationsresistenten Prostatakarzinoms (mCRPC). (*ADT* Androgendeprivationstherapie, *ARSI* Substanzen zur Blockade des Androgenrezeptorsignalweges, *RLT* Radioligandentherapie (Lutetium-PSMA), *nmHSPC/nmCRPC* nicht-metastasiertes mHSPC/mCRPC, *NA* unbekannt)

Bezüglich der eingesetzten Systemtherapien beim mHSPC, als auch bei der Erstlinientherapie des mCRPC zeigte sich ein signifikanter Unterschied zwischen den drei verglichenen Gruppen (Tab. 1). Die verschiedenen Therapiesequenzen der Gesamtperiode vom mHSPC hin zur Erstlinie des mCRPC sind in einem „sankey plot“ graphisch dargestellt (Abb. 2).

**Abb. 3:**
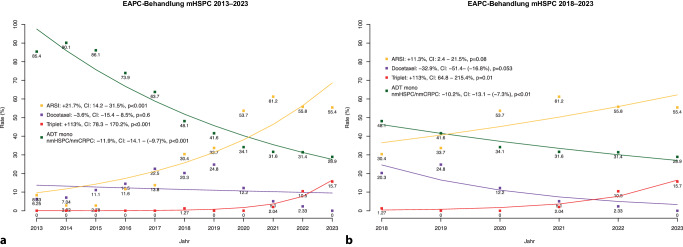
Trends der Systemtherapie („estimated annual percentage change“, EAPC) beim metastasierten hormonsensitiven Prostatakarzinom (mHSPC) im Zeitraum 2013–2023 (**a**) und 2018–2023 (**b**). (*ARSI* Substanzen zur Blockade des Androgenrezeptorsignalweges, *ADT* Androgendeprivationstherapie, *nmHSPC/nmCRPC* nicht-metastasiertes hormonsensibles/kastrationsresistentes Prostatakarzinom, *CI* Konfindenzintervall)

Bezüglich der EAPC-Analysen der letzten 10 Jahre der mHSPC-Patienten zeigt sich ein signifikanter Abfall der ADT-Monotherapie von 85 % im Jahr 2013 zu 29 % im Jahr 2023 beim mHSPC (EAPC: −11,9 %, *p* < 0,001; Abb. 3a). Umgekehrt zeigt sich ein signifikanter Anstieg der ARSI-Therapien von 6 % in 2013 auf 55 % in 2023 (EAPC: +21,7 %, *p* < 0,001). Hinsichtlich des Einsatzes der Docetaxel-Chemotherapie zeigt sich über die letzten 10 Jahre ein glockenhafter Verlauf mit einem 8 %-Einsatz in 2013 ansteigend auf bis zu 25 % im Jahr 2019 und einem Abfall auf 0 % in 2023.

Betrachtet man diese jährlichen Änderungsraten selektiver für die letzten 5 Jahre (2018–2023, Abb. 3b), so zeigt sich eine Änderung der Docetaxel-Chemotherapieanwendung von (20,3 % zu 0 %) zu Gunsten der ARSI-Therapie (30,4 % auf 55,4 %) sowie die Verabreichung einer Triplet-Therapie bestehend aus ARSI und Chemotherapie (bis zu 16 % in 2023).

## Diskussion

Die vorliegende Studie befasste sich mit der Fragestellung, inwieweit die Intensivierung der Kombinationstherapie beim mHSPC aus zahlreichen Phase-III-Studien Einzug in den alltäglichen klinischen Behandlungsalltag einer deutschen Metropolregion gefunden hat. Hierfür wurden entsprechende Stratifizierungen der mHSPC-Patienten gemäß dem Jahr der Metastasierungszeitpunkte, als auch verschiedene Trendanalysen durchgeführt und damit wichtige Erkenntnisse gewonnen.

Erstens zeigten sich klinisch wichtige, als auch statistisch signifikante Unterschiede in den Patienten- und Tumorcharakteristika der inkludierten mHSPC-Patienten nach Stratifizierung des Jahres der Metastasierungsdiagnose. Einerseits zeigte sich ein höheres Alter der medianen Metastasierung über die vergangenen Jahre hinweg (von 67 auf 71 Jahre), als auch eine Erhöhung des Anteils an De-novo-mHSPC-Patienten (von 48 % auf 62 %) in den Jahren ≤ 2006–2012 zu 2013–2017. Im Vergleich dazu zeigte sich keine signifikante Veränderung mehr in dem Verhältnis zwischen De-novo- und sekundär metastasierten mHSPC-Patienten in der letzten Dekade zwischen 2013–2023. Eine mögliche Erklärung für die entsprechend niedrigere relative Rate an sekundär metastasierten Patienten in der letzten Dekade im Vergleich zum Zeitraum ≤ 2006–2012 mag die Verbesserung des anatomischen Verständnisses und der damit verbundenen besseren onkologischen Resultate bei der radikalen Prostatektomie, als auch das bessere technische Verständnis und die Entwicklung der definitiven Strahlentherapie in der Behandlung des nicht-metastasierten Prostatakarzinoms sein [16–19]. Darüber hinaus steigt die Lebenserwartung von Männern in Deutschland weiter an, was zum Entstehen von De-novo-metastasierten PCa auch in höherem Alter führen könnte.

Weiterhin zeigt sich, dass aktuell behandelte mHSPC-Patienten einen tieferen absoluten PSA-Nadir aufwiesen, als Patienten die zwischen 2013–2017 sowie ≤ 2006–2012 behandelt wurden. Ebenso zeigt sich eine höhere Rate am relativen PSA-Abfall ≥ 99 % in den aktuell behandelnden mHSPC-Patienten. Dieser Trend ist von Bedeutung, da bereits mehrere Studien den Stellenwert des PSA-Nadirs als Surrogat auf das Gesamtüberleben nachweisen konnten. So zeigte beispielsweise eine zuletzt veröffentliche Post-hoc-Analyse der TITAN-Studie, dass bei mHSPC-Patienten ein Cut-off unter Apalutamid-Therapie ≤ 0,02 ng/ml mit den besten Überlebenswahrscheinlichkeiten vergesellschaftet ist [20, 21]. Dies ist als ein Hinweis auf die höhere Wirksamkeit der Kombinationstherapien zu werten, welche in der Zeit ebenfalls zugenommen haben.

Der Trend hin zu einem tieferen absoluten PSA-Nadir und der höheren Rate an relativen PSA-Abfall in unserer Kohorte deckt sich mit den gemachten Beobachtungen hinsichtlich der verwendeten Systemtherapie beim mHSPC. Die in den EAPC-Analysen dargestellten Kurven zeigen eine eindeutige Zunahme und Adaptation der intensivierten Systemtherapien beim mHSPC in der klinischen Alltagsrealität. Umgekehrt, zeigen die Analysen auch eine signifikante Abkehr von einer ADT-Monotherapie beim mHSPC innerhalb der letzten 10, als auch letzten 5 Jahren bis auf aktuell 29 %. Diese Trends sind insofern von Bedeutung, dass aktuelle Analysen anderer Arbeitsgruppen noch einen hohen Anteil an einer Nutzung der ADT-Monotherapie beim mHSPC u. a. auch in Deutschland von bis zu 34–58 % belegen [22, 23]. Die Zunahme der ARSI-Therapie und der drastische Abfall der Docetaxel-Chemotherapie innerhalb der letzten 5 Jahre zeigt die Adaption der ARSI-Kombinationstherapie und Triplet-Therapie als Goldstandard der Erstlinientherapie beim mHSPC. Ähnliche Szenarien und Trends aus anderen Ländern wurden in einem kürzlich veröffentlichen systematischen Review von Dodkins et al. bezüglich des mHSPC zusammengefasst [24]. Diese klinische Adaptation der intensivierten Therapie beim mHSPC der kürzlich publizierten Phase III ist wichtig, da die Kombinationstherapie aus ARSI zusätzlich zur ADT im Vergleich zur alleinigen ADT einen einem deutlichen absoluten als auch relativen Überlebensvorteil gezeigt haben [7, 8, 12]. Der Anstieg der Triplet-Therapie ist v. a. auf ausgewählte Patienten mit einer High-volume‑/High-risk-Erkrankung beschränkt, da hier in Studien die höchste Wirksamkeit nachgewiesen werden konnte [10, 13].

Die vorliegende Studie ist nicht frei von Limitationen. Neben dem monozentrischen Charakter der Studie sollte in der Interpretation der Daten auch die retrospektive Analytik Beachtung finden, welche mit den bekannten Limitationen wie nicht vollständig vorliegenden Patienten- und/oder Tumorcharakteristika von einzelnen Patientengruppen vergesellschaftet sein kann. Zum Beispiel konnte bei einigen Patienten der ADT-Monotherapien nicht zwischen einem mHSPC- oder einem nmCRPC- bzw. nmHSPC-Krankheitsstadium unterschieden werden. Alle Patienten mit ADT wurden jedoch als mHSPC-Patienten gewertet. Ebenso können Unterschiede in Stagingmodalitäten zu Verzerrungen geführt haben. Zudem spiegelt die aktuell vorliegende Studie die klinische Versorgungsrealität im Einzugsgebiet einer großen deutschen Metropolregion sowie einer Universitätsklinik nieder. Somit können die gewonnenen Erkenntnisse nicht auf alle deutschen Regionen und Behandlungsräume ausgeweitet werden.

## Fazit für die Praxis

- Die Studienergebnisse zeigen deutliche Veränderungen in den onkologischen vom metastasierten hormon-sensiblen Prostatakarzinom (mHSPC)-Patienten über die letzten Jahre hinweg, wie beispielsweise der absolute PSA-Abfall (prostataspezifisches Antigen).
- Zudem vor allem die Adaptation der ARSI-Therapie (Substanzen zur Blockade des Androgenrezeptorsignalweges) als Erstlinientherapie im mHSPC aus Phase-III-Studien sowie der Beginn der Verabreichung von sog. Triplet-Therapien im klinischen Alltag.
- Die ADT-Monotherapie (Androgendeprivationstherapie) wird erfreulicherweise nur noch seltener angewendet

## References

[1] Wala J, Nguyen P, Pomerantz M (2023) Early treatment intensification in metastatic hormone-sensitive prostate cancer. J Clin Oncol 41(20):3584–3590. 10.1200/JCO.23.00723 37267579 10.1200/JCO.23.00723 PMC10325768

[2] Tannock IF, de Wit R, Berry WR et al (2004) Docetaxel plus prednisone or mitoxantrone plus prednisone for advanced prostate cancer. N Engl J Med 351(15):1502–1512. 10.1056/NEJMoa040720 15470213 10.1056/NEJMoa040720

[3] de Bono JS, Logothetis CJ, Molina A et al (2011) Abiraterone and increased survival in metastatic prostate cancer. N Engl J Med 364(21):1995–2005. 10.1056/NEJMoa1014618 21612468 10.1056/NEJMoa1014618 PMC3471149

[4] Kyriakopoulos CE, Chen YH, Carducci MA et al (2018) Chemohormonal therapy in metastatic hormone-sensitive prostate cancer: long-term survival analysis of the randomized phase III E3805 CHAARTED trial. J Clin Oncol 36(11):1080–1087. 10.1200/JCO.2017.75.3657 29384722 10.1200/JCO.2017.75.3657 PMC5891129

[5] Fizazi K, Tran N, Fein L et al (2017) Abiraterone plus prednisone in metastatic, castration-sensitive prostate cancer. N Engl J Med 377(4):352–360 28578607 10.1056/NEJMoa1704174

[6] Wenzel M, Würnschimmel C, Nocera L et al (2021) Overall survival after systemic treatment in high-volume versus low-volume metastatic hormone-sensitive prostate cancer: systematic review and network meta-analysis. Eur Urol Focus. 10.1016/j.euf.2021.04.003 33853754 10.1016/j.euf.2021.04.003

[7] Chi KN, Agarwal N, Bjartell A et al (2019) Apalutamide for metastatic, castration-sensitive prostate cancer. N Engl J Med 381(1):13–24. 10.1056/NEJMoa1903307 31150574 10.1056/NEJMoa1903307

[8] Davis ID, Martin AJ, Stockler MR et al (2019) Enzalutamide with standard first-line therapy in metastatic prostate cancer. N Engl J Med 381(2):121–131. 10.1056/NEJMoa1903835 31157964 10.1056/NEJMoa1903835

[9] Mottet N, van Den Bergh R, Briers E, Cornford P, Santis MD, Fanti S et al (2019) EAU guidelines on prostate cancer. European Association of Urology, Arnhem

[10] Smith MR, Hussain M, Saad F et al (2022) Darolutamide and survival in metastatic, hormone-sensitive prostate cancer. N Engl J Med 386(12):1132–1142. 10.1056/NEJMoa2119115 35179323 10.1056/NEJMoa2119115 PMC9844551

[11] Fizazi K, Foulon S, Carles J et al (2022) Abiraterone plus prednisone added to androgen deprivation therapy and docetaxel in de novo metastatic castration-sensitive prostate cancer (PEACE-1): a multicentre, open-label, randomised, phase 3 study with a 2 × 2 factorial design. Lancet 399(10336):1695–1707. 10.1016/S0140-6736(22)00367-1 35405085 10.1016/S0140-6736(22)00367-1

[12] Mandel P, Hoeh B, Wenzel M et al (2022) Triplet or doublet therapy in metastatic hormone-sensitive prostate cancer patients: a systematic review and network meta-analysis. Eur Urol Focus 10.1016/j.euf.2022.08.007 36058809

[13] Hoeh B, Garcia CC, Wenzel M et al (2023) Triplet or doublet therapy in metastatic hormone-sensitive prostate cancer: updated network meta-analysis stratified by disease volume. Eur Urol Focus. 10.1016/j.euf.2023.03.024 37055323 10.1016/j.euf.2023.03.024

[14] Wenzel M, Nocera L, Ruvolo CC et al (2021) Racial/ethnic disparities in tumor characteristics and treatments in favorable and unfavorable intermediate risk prostate cancer. j Urol. 10.1097/JU.0000000000001695 33683934 10.1097/JU.0000000000001695

[15] Wenzel M, Ruvolo CC, Nocera L et al (2021) Regional differences in patient age and prostate cancer characteristics and rates of treatment modalities in favorable and unfavorable intermediate risk prostate cancer across United States SEER registries. Cancer Epidemiol 74:101994. 10.1016/j.canep.2021.101994 34364187 10.1016/j.canep.2021.101994

[16] Hoeh B, Wenzel M, Hohenhorst L et al (2021) Anatomical fundamentals and current surgical knowledge of prostate anatomy related to functional and oncological outcomes for robotic-assisted radical prostatectomy. Front Surg 8:825183. 10.3389/fsurg.2021.825183 35273992 10.3389/fsurg.2021.825183 PMC8901727

[17] Hatano K, Tohyama N, Kodama T et al (2019) Current status of intensity-modulated radiation therapy for prostate cancer: history, clinical results and future directions. Int J Urol. 10.1111/iju.14011 31115116 10.1111/iju.14011

[18] Numakura K, Kobayashi M, Muto Y, Sato H, Sekine Y, Sobu R, Aoyama Y, Takahashi Y, Okada S, Sasagawa H, Narita S, Kumagai S, Wada Y, Mori N, Habuchi T (2023) The Current Trend of Radiation Therapy for Patients with Localized Prostate Cancer. Curr Oncol 30(9):8092–8110. 10.3390/curroncol30090587. PMID: 37754502; PMCID: PMC10529045 37754502 10.3390/curroncol30090587 PMC10529045

[19] Andring LM, Abu-Gheida I, Bathala T, Yoder AK, Manzar GS, Maldonado JA, Frank SJ, Choi S, Nguyen QN, Hoffman K, McGuire SE, Mok H, Aparicio A, Chapin BF, Tang C (2023) Definitive local therapy for T4 prostate cancer associated with improved local control and survival. BJU Int 132(3):307–313. 10.1111/bju.16027. Epub 2023 Apr 25. PMID: 37057728 37057728 10.1111/bju.16027

[20] Hussain M, Tangen CM, Higano C et al (2006) Absolute prostate-specific antigen value after androgen deprivation is a strong independent predictor of survival in new metastatic prostate cancer: data from Southwest Oncology Group Trial 9346 (INT-0162). j Clin Oncol 24(24):3984–3990. 10.1200/JCO.2006.06.4246 16921051 10.1200/JCO.2006.06.4246

[21] Merseburger AS, Agarwal N, Bjartell A et al 1786P Effect of rapid ultra-low prostate-specific antigen decline (UL PSA) in TITAN patients (pts) with metastatic castration-sensitive prostate cancer (mCSPC) who received apalutamide (APA) plus androgen deprivation therapy (ADT). 10.1016/j.annonc.2023.09.2736

[22] Goebell PJ, Raina R, Chen S, Rege S, Shah R, Grossman JP, Waldeck AR (2024) Real-world treatment of metastatic hormone-sensitive prostate cancer in the USA, Europe and Asia. Future Oncol 20(14):903–918. 10.2217/fon-2023-0814. Epub 2024 Feb 14. PMID: 38353055 38353055 10.2217/fon-2023-0814

[23] Leith A, Ribbands A, Kim J et al (2022) Impact of next-generation hormonal agents on treatment patterns among patients with metastatic hormone-sensitive prostate cancer: a real-world study from the United States, five European countries and Japan. BMC Urol 22(1):33. 10.1186/s12894-022-00979-9 35277153 10.1186/s12894-022-00979-9 PMC8915525

[24] Dodkins J, Nossiter J, Cook A et al (2024) Does research from clinical trials in metastatic hormone-sensitive prostate cancer treatment translate into access to treatments for patients in the „real world“? A systematic review. Eur Urol Oncol 7(1):14–24. 10.1016/j.euo.2023.05.002 37380578 10.1016/j.euo.2023.05.002

